# Increased Arctic influence on the midlatitude flow during Scandinavian Blocking episodes

**DOI:** 10.1002/qj.3673

**Published:** 2019-11-19

**Authors:** Jonathan J. Day, Irina Sandu, Linus Magnusson, Mark J. Rodwell, Heather Lawrence, Niels Bormann, Thomas Jung

**Affiliations:** ^1^ European Centre for Medium‐Range Weather Forecasts Reading UK; ^2^ Alfred Wegener Institute, Helmholtz Centre for Polar and Marine Research Bremerhaven Germany

**Keywords:** Arctic, forecast error, observing system design, prediction, Scandinavian Blocking, teleconnections

## Abstract

Recent studies have suggested that Arctic teleconnections affect the weather of the midlatitudes on time‐scales relevant for medium‐range weather forecasting. In this study, we use several numerical experimentation approaches with a state‐of‐the‐art global operational numerical weather prediction system to investigate this idea further. Focusing on boreal winter, we investigate whether the influence of the Arctic on midlatitude weather, and the impact of the current Arctic observing system on the skill of medium‐range weather forecasts in the midlatitudes is more pronounced in certain flow regimes. Using so‐called Observing System Experiments, we demonstrate that removing *in situ* or satellite observations from the data assimilation system, used to create the initial conditions for the forecasts, deteriorates midlatitude synoptic forecast skill in the medium‐range, particularly over northern Asia. This deterioration is largest during Scandinavian Blocking episodes, during which: (a) error growth is enhanced in the European‐Arctic, as a result of increased baroclinicity in the region, and (b) high‐amplitude planetary waves allow errors to propagate from the Arctic into midlatitudes. The important role played by Scandinavian Blocking, in modulating the influence of the Arctic on midlatitudes, is also corroborated in relaxation experiments, and through a diagnostic analysis of the ERA5 reanalysis and reforecasts.

## Graphical abstract

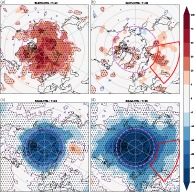

Change in RMS error (%) for *z*500 in the IN‐SITU OSE (top) and Arctic relaxation experiment (bottom) at a lead time of 1 day (T+24; left) and 4 days (T+96; right), with respect to the control for each experiment. Forecasts were started at 0000 and 1200 UTC between 1 December 2017 and 31 March 2018. The operational ECMWF HRES analysis is used to verify the forecast skill. The regions where (a,b) *in situ* data are denied and (c,d) relaxation is performed, are shown in magenta. The north Asia region is shown in red.

## 1 INTRODUCTION

In recent decades near‐surface air temperatures in the Arctic have warmed at approximately twice the global average (Serreze *et al*., 2009; Screen and Simmonds, 2010). The question of if, when, and how these changes in the Arctic environment will modify Northern Hemisphere circulation patterns has created widespread interest and is the subject of much debate (Cohen *et al*., 2014; Barnes and Screen, 2015). Some studies have suggested that Arctic amplification has already caused planetary‐scale waves to elongate meridionally and slow down, resulting in more frequent blocking patterns and extreme weather (e.g. Francis and Vavrus, 2012), while others find insufficient evidence for such conclusions (e.g. Barnes, 2013). As in other regions, it has been hard to robustly link the well‐known thermodynamic features of climate change, in this case Arctic amplification, to changes in atmospheric dynamics (Shepherd, 2014; Coumou *et al*., 2018).

A relevant question, and arguably one where more concrete progress can be made, is whether similar polar–midlatitude linkages play an important role for weather prediction (Jung *et al*., 2016). Recent studies have started to look at such teleconnections from a prediction perspective, concluding that improved Arctic prediction will lead to improved predictions at daily‐to‐seasonal ranges, particularly over northern parts of America and Asia (Jung *et al*., 2014). Indeed, it is thought that the tropospheric circulation over north Asia is more closely tied to the circulation in the Arctic than the Tropics (Ye *et al*., 2018). This is thought to be because the mean stationary‐wave structures in the Northern Hemisphere promote flow out of the Arctic into north Asia (Semmler *et al*., 2018).

The evidence gathered so far on how improvements in weather predictions over the Arctic could lead to improvements in midlatitudes relies on two types of numerical experimentation. The first type of experiments, so‐called relaxation experiments (Jung *et al*., 2014), consist of a weather forecast in which the state variables in the Arctic are relaxed (or nudged) toward an atmospheric analysis (or reanalysis, such as ERA‐Interim or ERA5: Dee *et al*., 2011; Hersbach *et al*., 2019) throughout the entire forecast range. Analyses (or reanalyses) produced with modern numerical weather prediction (NWP) systems represent the best available reconstruction of the present (past) atmospheric state, obtained by blending a forecast model and observations through the data assimilation process. In the relaxation experiments, the Arctic is therefore “perfectly” represented throughout the forecast range. By comparing the relaxation experiment with the normal free‐running forecasts, one obtains an upper bound to the forecast skill improvement that could be achieved in midlatitudes by improving the quality of the forecasts in the Arctic.

The second type of experiments are so‐called Observing System Experiments (OSEs), in which specific observations are not assimilated (or are denied) when creating the initial conditions for weather forecasts. The accuracy of weather forecasts depends on the three key ingredients of a modern NWP system (Bauer *et al*., 2015): forecast model, data assimilation methods and observations, which are used in the data assimilation process to create the initial conditions for each forecast (the analysis). As the usage of observations to produce an accurate initial state is crucial for a skilful forecast, denying Arctic observations provides another type of numerical experiment that can be used to investigate how improved initial conditions over the Arctic could lead to improved forecasts in midlatitudes. Such experiments can also be used to quantify the impact of specific Arctic observations on the initial conditions in the Arctic, and subsequently on the forecast skill in the midlatitudes. However, until recently such OSEs have only been performed for Arctic observations in a reduced complexity forecasting system (Sato *et al*., 2018) and have tended to focus on specific case‐studies, such as cold air outbreaks (Sato *et al*., 2017). As a result, they are not necessarily representative of what one might find in a comprehensive operational global NWP system, such as the Integrated Forecasting System (IFS) of the European Centre for Medium‐range Weather Forecasts (ECMWF).

OSEs were recently performed to assess the impact on forecast skill of different observation types in the Arctic using the ECMWF IFS. Experiments removing various groups of Arctic observations (including “*in situ*” observations – from aircraft and radiosonde, etc. – and a wide range of satellite observations), north of 60°N, were run for both a winter and a summer season. A thorough assessment of the impacts of different Arctic observation types over both seasons is presented by Lawrence *et al*. (2019). Results show the importance of all observations in reducing forecast error in the Arctic, with the highest impacts due to microwave radiances in summer and *in situ* observations in winter. The midlatitude region most impacted by denying Arctic observations during winter was found to be northern Asia, which is consistent with the results of previous studies which used relaxation experiments to explore Arctic‐to‐midlatitude linkages (Jung *et al*., 2014; Semmler *et al*., 2018).

In this article we use both relaxation and OSE experiments, with the aim to better understand whether the influence of the Arctic on medium‐range weather forecasts depends on the flow situation (the prevailing flow “regime”). This aspect was not examined in Lawrence *et al*. (2019). Specifically, we want to understand (a) whether the Arctic has an influence on the skill of medium‐range weather forecasts in northern Asia in all cases, or whether the strength of the influence varies as a result of changes in flow regime; and (b) whether insights about the regime dependence of the influence of the Arctic on the midlatitudes, gained from the Arctic OSEs, are consistent with those derived from relaxation experiments. To answer these questions, we will compare the regime‐dependence of results from OSEs for the Arctic region with results from a set of relaxation experiments run for the same period, using the methodology of Jung *et al*. (2014).

The two approaches used, relaxation experiments and the OSEs, are of course conceptually very different. The OSEs investigate the impact of locally degraded initial conditions, obtained by denying observations from the assimilation system in the Arctic. The forecast degradation in these experiments therefore arises only from the degraded initial conditions. The nature of the degradation in the initial conditions depends, among other things, on the coverage of the denied observing system and our ability to assimilate the observations successfully. In contrast, the relaxation experiments suppress forecast error growth in the Arctic during the forecast integration, and this covers growth of errors in the initial conditions as well as errors arising from deficiencies in the representation of physical processes in the forecast model. For a given forecast lead time, the origin of the forecast degradation in the OSEs on the one hand and the forecast improvement in the relaxation experiment on the other may hence be very different. However, both approaches enable us to investigate under which regimes forecast skill in the midlatitudes is more sensitive to the Arctic region. Investigating whether the two methods indicate similar regimes is one of the aims of this article.

To further investigate whether and how forecast error growth in the Arctic and northern Asia depends on specific flow regimes we also perform a diagnostic analysis of the ERA5 reanalysis and reforecasts (Hersbach *et al*., 2019). This allows us to corroborate the insights into the regime dependence of Arctic‐to‐midlatitude linkages gained from the OSEs and the relaxation experiments, which are limited to one particular season, over a longer period. Combining experimental approaches, such as relaxation, and diagnostic methods like this has been useful in diagnosing the sources of forecast error (Magnusson, 2017), and may provide further insight than a single approach.

## 2 NUMERICAL EXPERIMENTATION

### 2.1 Observing system experiments

The OSEs described in this study were carried out with the operational version of the ECMWF IFS (cycle 45r1 was used in operations from July 2018 to June 2019), at a horizontal resolution of TCo399 (≈25 km), which is lower than the resolution of the ECMWF operational high‐resolution deterministic analyses and 10‐day forecasts (operational HRES hereafter, ∼9 km). The vertical resolution is the same as in the operational HRES, i.e. 137 levels.

As in the operational HRES, the incremental 4D‐Variational (4D‐Var) data assimilation system (Rabier *et al*., 2000; Bonavita *et al*., 2012) is used to produce analyses by assimilating satellite and *in situ* observations in two 12 h assimilation windows (0900 UTC to 2100 UTC and 2100 UTC to 0900 UTC). The analyses are then used as initial conditions for 10‐day weather forecasts initialized each day at 0000 and 1200 UTC respectively.

In the OSEs, various observations were removed from the 4D‐Var in the Arctic and Antarctic (above 60°N and below 60°S) in order to investigate the importance of the polar observations. In this study, we focus on the OSEs performed for the period December 2017–March 2018 (DJFM 2017/2018) and on the role of Arctic observations. This period contains the Year of Polar Prediction (YOPP) First Special Observing Period (SOP1), February–March 2018, during which many of the Arctic radiosonde stations doubled or tripled the frequency of radiosonde launches (Goessling *et al*., 2016). Apart from the Sudden Stratospheric Warming event which occurred around mid‐February (Karpechko *et al*., 2018) this season does not stand out from a synoptic point of view. Indeed, the distribution of Euro‐Atlantic regimes is quite close to climatology (not shown).

The comprehensive set of OSEs performed for DJFM 2017/2018 is described in detail in Lawrence *et al*. (2019). In this study, we mainly focus on the three OSEs which showed the largest impact on medium‐range forecast skill in the midlatitudes, and which are therefore the most relevant for evaluating the behaviour of Arctic–midlatitude linkages. These are the OSEs in which the following observations were removed:

1. All *in situ* observations (IN‐SITU hereafter)
2. All microwave radiance observations (MW hereafter), from AMSU‐A (6 instruments), ATMS (1 instrument), MHS (4 instruments), GMI (1 instrument, 183 GHz humidity‐sounding channels only), SSMI/S (2 instruments, 183 GHz humidity‐sounding channels only), MWHS (1 instrument), and MWHS‐2 (1 instrument),
3. All infrared radiance observations (IR hereafter), from IASI (2 instruments), CrIS (1 instrument), and AIRS (1 instrument)

However, to better understand which specific observation type is having the largest impact for specific cases, we also make use of other OSEs discussed in Lawrence *et al*. (2019) in which only (a) radiosondes, (b) surface pressure observations, (c) the extra radiosondes associated with YOPP‐SOP1, (d) temperature‐sensitive MW channels and (e) humidity‐sensitive MW channels were removed.

The forecast errors in the OSEs were compared to those in a control experiment, run for the entire period, in which all observations were assimilated globally.

### 2.2 Relaxation experiments

A relaxation experiment was carried out with the ECMWF IFS for the same period as the OSEs, i.e. DJFM 2017/2018. This experiment consists of 10‐day weather forecasts initialized each day at 0000 and 1200 UTC from the ECMWF operational analyses, in which the relaxation methodology described in Jung *et al*. (2010), Jung (2011), and Hoskins *et al*. (2012) is applied. In this approach, the model forecast is relaxed toward the operational analysis throughout the forecast in order to reduce forecast errors in the Arctic. This is done by modifying the temporal evolution of the model according to the following equation:

$$\partial x/\partial t=F\left(x\right)-\alpha \left(x-x_{\mathrm{ana}}\right)$$

where *x* denotes the model state vector, *α* denotes the relaxation coefficient, *x*_ana_ denotes the ECMWF operational analysis respectively and *F(x)* represents the model's prognostic equations. The value of *α* controls the strength of the relaxation and is set to 1/3 (per time step) above 75°N. This means that at each time step the model is corrected using 1/3 of the departure of *x* from *x*_ana_ for all variables and levels. To achieve a smooth transition between the relaxed and free running regions, *α* is smoothed between 75°N and 65°N by an exponential function that approaches 0N at 65°N. Note that the southernmost extent of the relaxation zone, 65°N, is slightly further north than the 60°N cut‐off used in the OSEs. The choice of the value of *α* is based on the consideration of both the strength of the relaxation and the model stability. The relaxation of the atmospheric variables is carried out by interpolating from the 6 h ECMWF operational analyses (*x*_ana_) to the actual time step. The atmospheric state variables: surface pressure, temperature, humidity and horizontal winds are relaxed at each model time step throughout the 10‐day forecasts.

Changes in forecast error in the relaxation experiment were compared to those in a control experiment without relaxation, run for the same period. These experiments were performed with cycle 43r3 of the ECMWF IFS (used in operations from July 2017 to July 2018) at a horizontal resolution of TCo639 (≈18 km) with 137 levels in the vertical. The relaxation experiments use a slightly older version of the IFS and a higher spatial resolution than the OSEs, but these differences in the model version and resolution with respect to the OSEs are not expected to affect the interpretation of the outcomes.

As in previous studies, the relaxation approach is used to determine whether improving the representation of the atmospheric circulation in the Arctic would improve the skill in the midlatitudes. In this study, we use this technique to see whether we get similar insights about the regime dependence of the Arctic's influence on the midlatitudes compared to OSEs.

### 2.3 Skill metrics and significance testing

To express the change in skill between the OSEs and relaxation experiments compared to the control run, we use the normalized root‐mean‐square error (RMSE_expt_−RMSE_ctrl_)/RMSE_ctrl_), multiplied by 100 to arrive at the percentage change in forecast error. Throughout the text we will refer to this as the change in RMSE. A paired *t*‐test is used to calculate a 95% significance threshold. In order to account for temporal autocorrelation the error bars are inflated by 1.22 and an additional “Sidak correction” is applied to further inflate the error bars to account for the multiple tests (i.e. multiple OSEs), which would otherwise increase the chances of a false positive (Geer, 2016). All experiments are verified against the operational ECMWF high‐resolution analysis.

## 3 ARCTIC TO MIDLATITUDE LINKAGES FROM OSES AND RELAXATION EXPERIMENTS

### 3.1 Impact on medium‐range forecast skill

**Figure 1:**
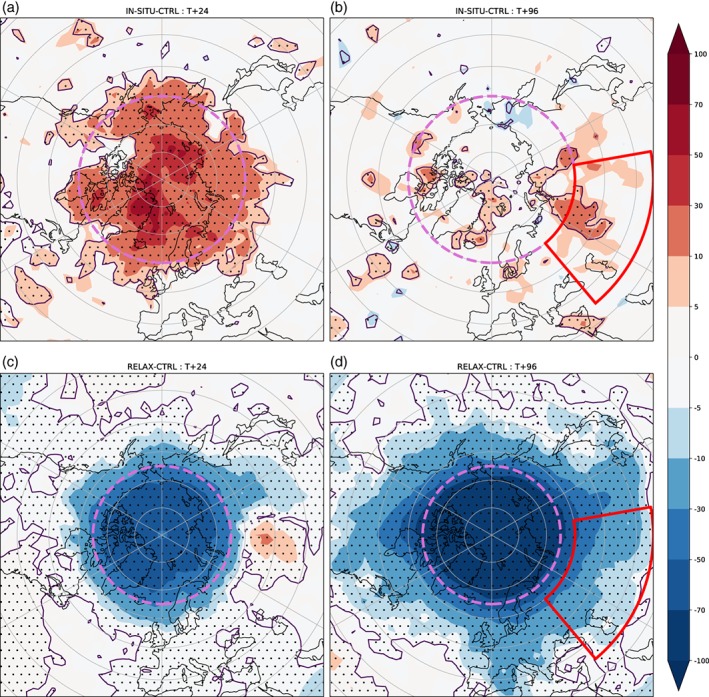
Change in RMS error (%) for *z*500 in the (a,b) IN‐SITU OSE and (c,d) Arctic relaxation experiment, at a lead time of (a,c) 1 day (T+24) and (b,d) 4 days (T+96), with respect to the control for each experiment. Forecasts were started at 0000 and 1200 UTC between 1 December 2017 and 31 March 2018. The operational ECMWF HRES analysis is used to verify the forecast skill. The region where *in situ* data are denied (a,b) and relaxation is performed (c,d) is shown in magenta. The North Asia region is shown in red [Colour figure can be viewed at http://wileyonlinelibrary.com].

**Figure 2:**
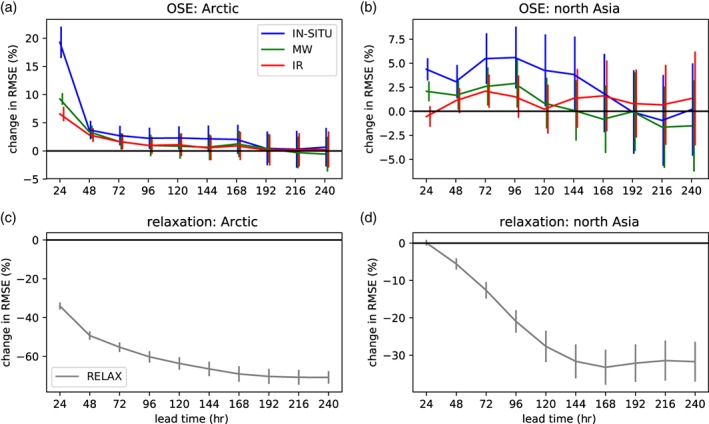
(a) Change in *z*500 RMS error (%) in the Arctic (>60°N) for the IN‐SITU, MW and IR OSEs with respect to the control experiment, shown as a function of lead‐time; (b) as (a) but for the north Asia region shown in Figure 1; (c,d) as (a,b) but for the relaxation experiment [Colour figure can be viewed at http://wileyonlinelibrary.com].

As discussed by Lawrence *et al*. (2019), the observing system which has the largest impact on forecast skill both in the Arctic and in the midlatitudes during winter is the conventional *in situ* observations. At short lead times (day 1), the largest impact is within the Arctic itself, where the IN‐SITU OSE increases the error in geopotential height at 500 hPa (*z*500) compared to the control experiment (Figures 1a and 2a). Note that verification against operational analyses for short‐range forecasts is problematic as analysis errors are comparable to short‐range forecast errors both in magnitude and characteristics, so the magnitude of the reduction in RMSE should be treated with some caution (Lawrence *et al*., 2019). Despite this caveat, we show the change in RMSE at day 1 in Figures 1 and 2 to highlight the fact that the differences between the IN‐SITU OSE and the control are largest in the Arctic at short lead times.

In the midlatitudes, the impact becomes visible at longer lead times (Figures 1b and 2b), after the signal induced by the change of the initial conditions in the Arctic has had time to propagate. In northern Asia, which is the region of the midlatitudes that exhibits the largest increase in RMSE at day 4 (indicated by the box in Figure 1b, hereafter referred to as “north Asia”), the reduction in skill in the IN‐SITU OSE is significant until day 5, but peaks at days 3–4 with values of about 6% (Figure 2b). Interestingly, the decreases in skill in the IR and MW OSEs also peak at days 3–4 (Figure 2b). This suggests that this is the average time it takes for the signal to reach north Asia, regardless of the observation type that has been removed from the Arctic when creating the initial conditions of the forecasts.

To put these changes in skill into context, the performance of the ECMWF operational medium‐range weather forecasts improves at a rate of approximately 2% per year in key metrics such as *z*500 RMSE in the Northern Hemisphere (Geer, * * 2016), due to combined changes to all components of the NWP system (Bauer *et al*., 2015).

North Asia is a region that is also significantly affected in the relaxation experiment (Figure 1c,d), likely because the northerly component of the mean stationary waves are strongest there (Semmler *et al*., 2018). For this type of experiment, Arctic influence appears as a reduction in forecast error. While there are some similarities with the OSEs in terms of the broad area, there are clear differences in both the strength and meridional extent of the influence, with the relaxation experiment having a stronger impact over a wider area than the OSE. In the relaxation experiments, the magnitude of the change in skill is much larger, and lasts throughout the forecast (Figure 2c,d), because the forecast is continually “relaxed” back to the analysis state in the Arctic. In contrast, in the OSEs it is only the quality of the initial conditions which is degraded. The largest relative improvements in the relaxation experiments are obtained later in the forecast (Figure 2c,d). For the Arctic region itself, this is because the strength of the relaxation increases linearly with the error, and so the relaxation strongly reduces the asymptotic limit of the RMSE (which otherwise continues to grow in the control forecast).

Outside the region of relaxation, the reduced errors may reflect causal links in the real atmosphere from the Arctic into the midlatitudes, but one should keep in mind that these could be over‐emphasized if the relaxation term represents a strong forcing on e.g. planetary waves (so that an Arctic “tail” could be wagging a midlatitude “dog” to some extent). In contrast, the differences in errors between the OSEs and the control tend to diminish with lead‐time (Figure 2a,b) and become less spatially coherent and fade out through being overwhelmed by midlatitude dynamics. This may also be due to the overestimation of the impact in the short‐range discussed above, and the increasing impact of a limited sample‐size, respectively. These different caveats mean that the two techniques can be viewed as complementary.

Previous relaxation experiments have also highlighted North America as one of the regions that would benefit the most from improved Arctic forecasts (Jung *et al*., 2014), again due to the location of mean stationary waves (Semmler *et al*., 2018). While our relaxation experiments corroborate this result, forecast skill in the medium‐range is however not significantly affected over North America in the IN‐SITU (see Figure 1), MW or IR (not shown) OSEs. This may suggest that initial conditions over the Arctic are not the main factor for the Arctic influence over North America found in the relaxation experiments. This aspect could be investigated further, including the regime dependence of the influence over North America, but this is considered beyond the scope of this study.

Clearly, there is not a one‐to‐one relationship between the regions where forecast skill is affected in these two approaches, or in the magnitude of the change. Nevertheless, we are predominantly interested in determining when and how the Arctic has an influence, and we look to assess the regime dependence of the Arctic influence on north Asia using both methods in the following section.

### 3.2 Regime dependence

In this section, we investigate if the degradation ( improvement) in medium‐range forecast skill over north Asia obtained in the OSEs (relaxation experiments) is related to specific flow regimes. As in the previous section, we focus our analysis on the north Asia region (indicated in Figure 1, 40°E–100°E, 35°N–60°N) where the largest change in skill is found at day 4 in the OSEs. This region is very similar to that highlighted in previous studies which have used relaxation experiments to explore Arctic to midlatitude linkages (e.g. Jung *et al*., 2014; Semmler *et al*., 2018).

**Figure 3:**
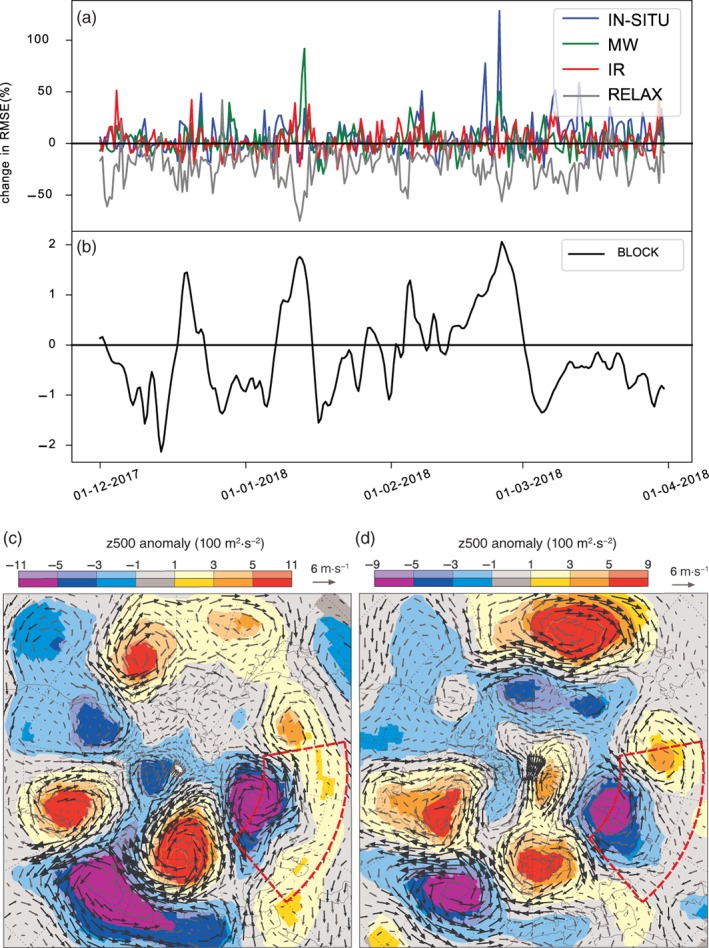
(a) Time series of the change in *z*500 RMSE (%) at day 4 (in the north Asia box shown in Figure 1) for the IN‐SITU, MW and IR OSEs and relaxation experiment, with respect to the control experiments. (b) The Scandinavian blocking index. (c) The difference in *z*500 initial conditions for cases where the OSEs have a high impact minus those with low impact, selected based on the change in *z*500 RMSE in north Asia (shown in red) at day 4. First the maximum change in RMSE in the IN‐SITU, MW and IR OSEs with respect to the control is calculated, and the dates with the highest (lowest) 50% of this quantity are included in the high error (low error) set of dates. (d) Difference in *z*500 initial condition for relaxation high‐impact minus low‐impact cases, selected as in (a) but based on the change in RMSE in north Asia in the relaxation experiment [Colour figure can be viewed at http://wileyonlinelibrary.com].

The time series of changes in *z*500 RMSE at day 4 in this region, with respect to the control, reveals that the impact of removing the observations in the different OSEs varies significantly between consecutive forecasts (Figure 3a). Although on average the forecast skill is degraded in this region in each of the OSEs (Figure 2), because of the chaotic nature of the atmosphere, forecasts initialised on certain dates can be slightly improved. Others are severely degraded, with the RMSE increasing by more than 100% in certain OSEs with respect to the control (Figure 3). The observation type, which has the largest impact, varies from day to day. However, there are distinct clusters of forecasts where in general the observations seem to be having a larger impact and periods where there is little impact from any of the observation types.

The impact of Arctic relaxation also varies in time (Figure 3a) but the periods during which the skill is affected in this region are more continuous than for the OSEs. The clusters of forecast start dates where denying observations leads to a reduction in skill approximately mirror the clusters where the relaxation experiment leads to the largest improvement in skill in north Asia (Figure 3a). The question arises as to whether both the relaxation experiment and the OSEs are only having an impact on this region when the forecast is initialised during a particular flow regime.

It can be seen that over the entire season, the change in *z*500 RMSE between the three OSEs and their control and the change in *z*500 RMSE between the relaxation experiment and its control, is highest during periods where the Scandinavian Blocking index is also high (Figure 3b). This index is calculated as the normalized dot product between the *z*500 anomaly field for each time (from the ERA‐Interim reanalysis) and a pre‐defined Scandinavian Blocking pattern (also expressed as a *z*500 anomaly). The Scandinavian Blocking pattern we used is one of the four standard Euro‐Atlantic regimes used in ECMWF products and was originally computed using a “k‐means” clustering algorithm, based on daily *z*500 reanalysis fields (further details can be found in Ferranti and Corti, 2011; Ferranti *et al*., 2015).

To confirm the hypothesis that the Scandinavian Blocking pattern characterizes the flow during periods when the OSEs lead to an impact over north Asia, we select the cases for which the OSEs have the largest impact over that region. To do this, we construct the time series of the maximum change in *z*500 RMSE obtained at day 4 in north Asia, across the three OSEs (IN‐SITU, MW, IR) with respect to the control. Then we select the forecast start times for which this metric is high (above 50th percentile) and low (below 50th percentile), respectively. We then plot the difference in the mean *z*500 field (from the initial analysis) between the cases in which overall impact of the OSEs on forecast skill in this region is higher or lower than average (Figure 3c). We also construct a similar composite for the impact of the relaxation at day 4 in the same region (Figure 3d). The high/low impact in this case is measured in terms of the change in RMSE in the relaxation experiment with respect to the control.

From this composite, it can be clearly seen that the largest impacts at day 4 over the north Asia box, both in the OSEs and relaxation experiments, tend to occur in forecasts initialised during conditions characterized by a Scandinavian Blocking regime (Figure 3c,d). The *z*500 anomalies associated with these periods of increased Arctic influence are qualitatively quite similar to those shown in Semmler *et al*. (2018, see their figures 5 and 6), which also suggests that the Arctic has more influence on the midlatitudes during periods of northerly flow associated with cold air outbreaks over Asia.

It is well known that forecast errors can propagate along planetary waves at the group velocity (Kelly *et al*., 2007; Magnusson, 2017). Indeed, one might expect that a regime, such as that shown in Figure 3c,d, with high pressure over Scandinavia and low pressure over northern Siberia, would lead to the propagation of forecast errors out of the Arctic and into our region of interest. However, we will further investigate this type of error propagation by selecting specific case‐studies.

### 3.3 Case‐studies

**Figure 4:**
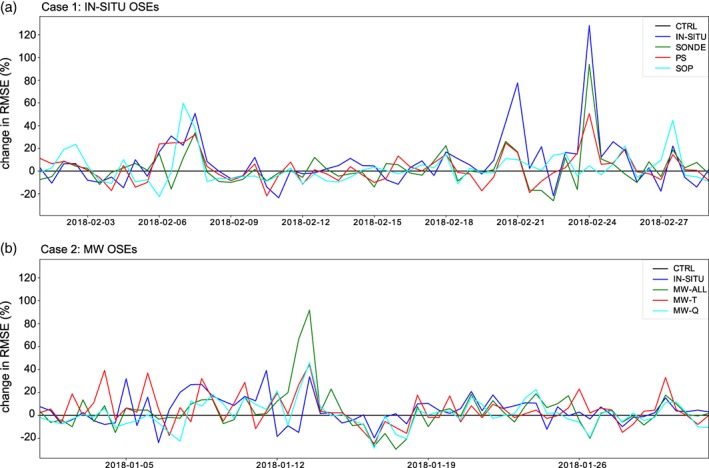
(a) Time series of the change in *z*500 RMSE (%) at day 4 (in the north Asia box shown in Figure 1) for the IN‐SITU, radiosonde, surface pressure and SOP1 OSEs with respect to the control experiment for February 2018. Initialisation time is shown. (b) As (a) but for the IN‐SITU, all‐microwave, microwave‐temperature and microwave‐humidity OSEs with respect to the control for January 2018 [Colour figure can be viewed at http://wileyonlinelibrary.com].

In order to understand how the Scandinavian Blocking regulates the influence of the Arctic on north Asia, we investigate the impact of Arctic observations and relaxation on forecast errors in two cases. To do this we selected the cases where denying Arctic observations had the highest and second highest impact on forecast performance over the north Asia box at day 4 (Figures 3 and 4).

The forecast with the highest impact was initialised on 24 February 2018, with the IN‐SITU OSE resulting in the largest degradation in skill. For this case, the forecast was also severely degraded even when only the radiosondes, or only the surface pressure observations, were removed from the initial conditions (a 75 and 46% increase in *z*500 RMSE respectively; see Figure 4). The second case‐study focuses on the second largest deterioration in forecast performance in the OSEs which occurred on 13 January 2018 at 1200 UTC (Figures 3 and 4b). In this case, the largest impact was obtained by denying the MW observations. In Figure 4b, it can be seen that both temperature‐sensitive and humidity‐sensitive microwave channels, as well as *in situ* observations, had an impact on the forecast.

**Figure 5:**
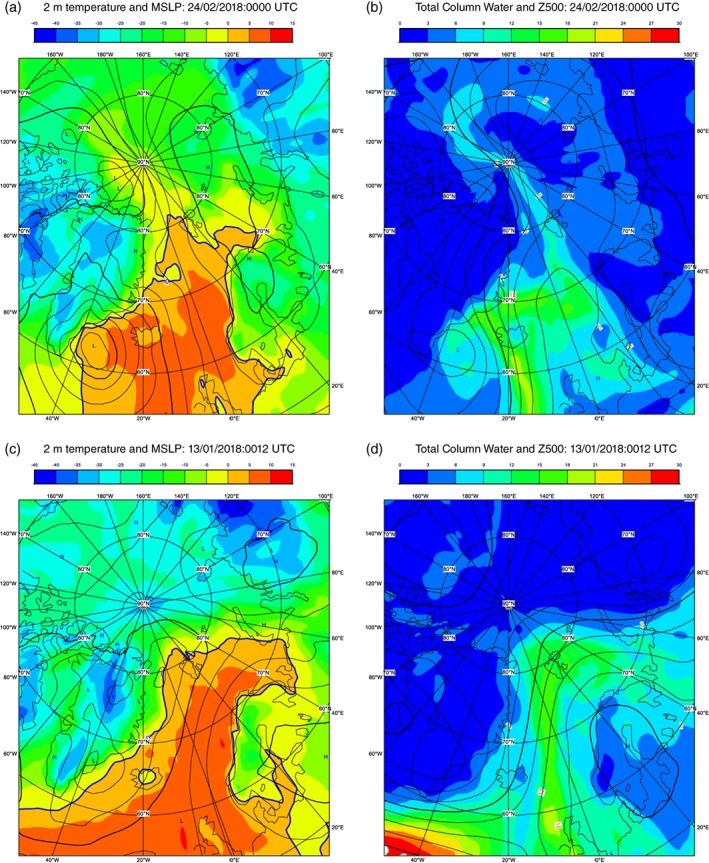
(a) 2 m temperature (filled contours) and mslp (isolines) and (b) total column water vapour (filled contours) and *z*500 (isolines), from the operational analysis for 24 February 2018, 0000 UTC. (c,d) As (a,b) except for 13 January 2018, 1200 UTC [Colour figure can be viewed at http://wileyonlinelibrary.com].

The meteorological situation at the initial time of both cases was characterized by a warm‐moist intrusion into the Arctic, associated with a ridge of high pressure extending into the Arctic (Figure 5). In both cases the situation bears a strong resemblance to the December 2015 extreme Arctic warm event described in Binder *et al*. (2017), which was characterized by a low‐pressure centre in the lee of Greenland and high pressure over Europe (see their figure 4). In this situation, they argue that air with low values of potential vorticity (PV) from low latitudes is transported along a warm conveyor belt (WCB) into high latitudes, to form and maintain the ridge.

The 24 February 2018 case coincided with the Sudden Stratospheric Warming event. The meteorological situation was characterized by a period of exceptional warming in the Arctic, with a ridge and associated warm‐anomaly extending high into the Arctic. The pressure pattern on 13 January 2018 is more typical of a period of Scandinavian Blocking with the region of high pressure confined to northern Europe. Although there are some differences, the cases are quite similar to each other and to other Arctic warm episodes, such as those described in Moore (2016) and Messori *et al*. (2017).

It is also interesting to note that in both cases the impact of midlatitudes on the Arctic also seems to be strong. For example, the air‐mass trajectory analysis of Binder *et al*. (2017) showed that air masses in the high Arctic during such episodes originated as far south as the Sahara.

**Figure 6:**
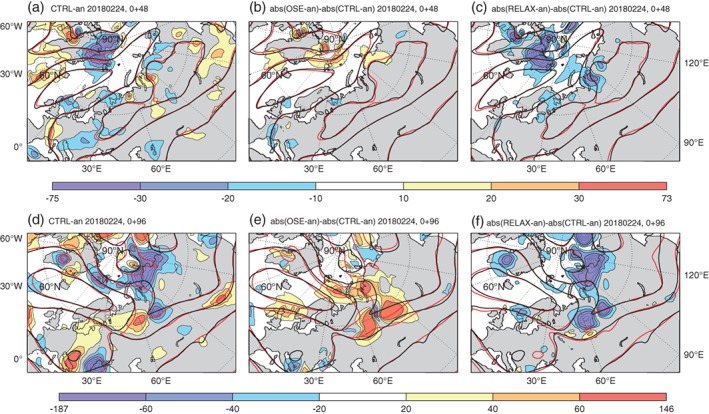
Evolution of *z*500 errors in control forecast compared to the operational analysis (started 24 February 2018, 0000 UTC; a,d), the difference in absolute error for the IN‐SITU OSE compared to the control (filled contours, b,e), the same for the relaxation experiment compared to the control (filled contours, c,f). Line contours show the actual *z*500 field from the operational analysis (black) and the control forecast (a,d), the OSE (b,e) and the relaxation experiment (c,f), all in red. All panels in the top row are at T + 48 and the bottom are at T+96 [Colour figure can be viewed at http://wileyonlinelibrary.com].

Examining the *z*500 errors in the forecast initialised on 24 February 2018, it appears that by day 2 of the control forecast, errors in *z*500 (i.e. the difference in black and red contours, or the coloured shading) have grown most rapidly around a developing trough north of Greenland (Figure 6a). These errors are larger in the IN‐SITU OSE (Figure 6b) and smaller in the relaxation experiment (Figure 6c), as one would expect.

By day 4 of the forecast, a closed‐contour is evident in the Arctic and the cyclone has moved across the Arctic toward the Siberian coastline and the ridge has started to decay. The control forecast has a region of *z*500 values which are too low on the landward side of the cyclone compared to the analysis, suggesting that errors are growing rapidly in this region associated with moist processes in the WCB (Figure 6d). These errors (i.e. the difference between black and red contours in Figure 6b,c,e,f) are much larger in the IN‐SITU OSE than the control (i.e. shading shows mainly positive values in Figure 6b,e), but as expected they are reduced in the relaxation experiment (i.e. shading shows mainly negative values in Figure 6c,f).

**Figure 7:**
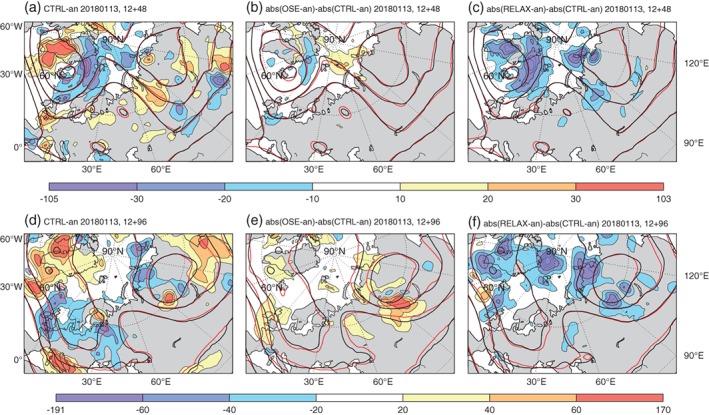
As Figure 6, but for forecast experiments started on 13 January 2018, 1200 UTC [Colour figure can be viewed at http://wileyonlinelibrary.com].

By day 2 of the control forecast initialised on 13 January 2018, errors in *z*500 have grown most rapidly around the cyclone located to the east of Greenland (Figure 7a). There are negative *z*500 errors in the warm sector and positive errors in the cold sector, suggesting that either the air in the cyclone's warm/cold sector is too cold and dry/warm and humid, resulting in a ridge which is too weak. This ridge is even weaker in the MW OSE (Figure 7b), but the error is corrected in the relaxation experiment (Figure 7c), as one would expect.

By day 4, a distinct region with increased *z*500 error can be seen in the MW OSE over north Asia (Figure 7e), where the additional errors introduced in the Arctic at the beginning of the forecast have grown. A corresponding reduction of errors over north Asia is seen in the relaxation experiment, confirming that this error has an Arctic origin (Figure 7f).

In both cases, the inclusion of data denied in the OSEs and the relaxation to analysis improve forecast skill both within the Arctic, and over north Asia. Errors in the forecasts are growing much more rapidly in regions where moist processes are important, i.e. along the WCBs associated with the cyclones (in Figures 6a and 7a,d) and subsequently these are the regions where additional Arctic observations and relaxation have the largest impact.

It is well known that forecasts' errors grow very rapidly along WCBs in such situations (Rodwell *et al*., 2018). Under this type of flow configuration, when the WCB will be advecting air with low PV into higher latitudes (Binder *et al*., 2017), moist processes, such as the release of latent heat in ascending air streams within the WCB, may also be important for the evolution of the ridge itself (Pfahl *et al*., 2015). This provides a mechanism for enhanced initial errors in the formation of the ridge, in the OSEs, to influence and degrade the forecast of the trough formation downstream, over north Asia, at later lead times (Figures 6e and 7e).

## 4 CLIMATOLOGICAL FORECAST ERROR GROWTH DURING SCANDINAVIAN BLOCKING EPISODES

Analysis of the observing system and relaxation experiments suggested that forecast errors, or corrections, introduced in the Arctic have the largest effects on the weather in the midlatitudes during episodes when errors can propagate out of the Arctic along planetary waves. In particular, enhanced errors in the European Arctic sector, during Scandinavian Blocking episodes, were shown to impact skill over north Asia. One might wonder what is so important about the Arctic observations during this regime. One hypothesis, suggested by the two case‐studies, is that short‐range forecast errors are simply larger in the Arctic during forecasts initialised during situations characterized by Scandinavian Blocking. If so, removing observations in this region, when creating the initial conditions (in the case of the OSEs), will have a larger impact than during other periods.

To test this, we calculate climatological forecast errors from the ERA5 reanalysis and reforecasts (Hersbach *et al*., 2019) for several recent winter seasons (2013–2018). We use ERA5 instead of the ECMWF operational HRES analysis, to look at a long period with a consistent forecasting system (ERA5 is based on Cy41r2 of the ECMWF IFS, operational between March and November 2016).

This analysis of short‐range forecast errors builds on previous work on regime‐dependent predictability in the late‐medium‐range, which has shown that ECMWF forecasts initialised during a Scandinavian Blocking situation tend to have lower skill in the Euro‐Atlantic region than those initialised during other regimes due to a lack of persistence of the Scandinavian Blocking regime (Ferranti *et al*., 2015). Low forecast skill is also often associated with the onset of blocking (Rodwell *et al*., 2013). The case‐study analysis by Grams *et al*. (2018) suggests that errors develop along WCBs because of errors in diabatic processes. Such WCBs transport anomalous‐PV air poleward and such errors in diabatic heating are a cause of errors in the large‐scale wave structure. Clearly, if the WCB outflow region is positioned inside the Arctic, during a given event, this should lead to higher error growth there.

**Figure 8:**
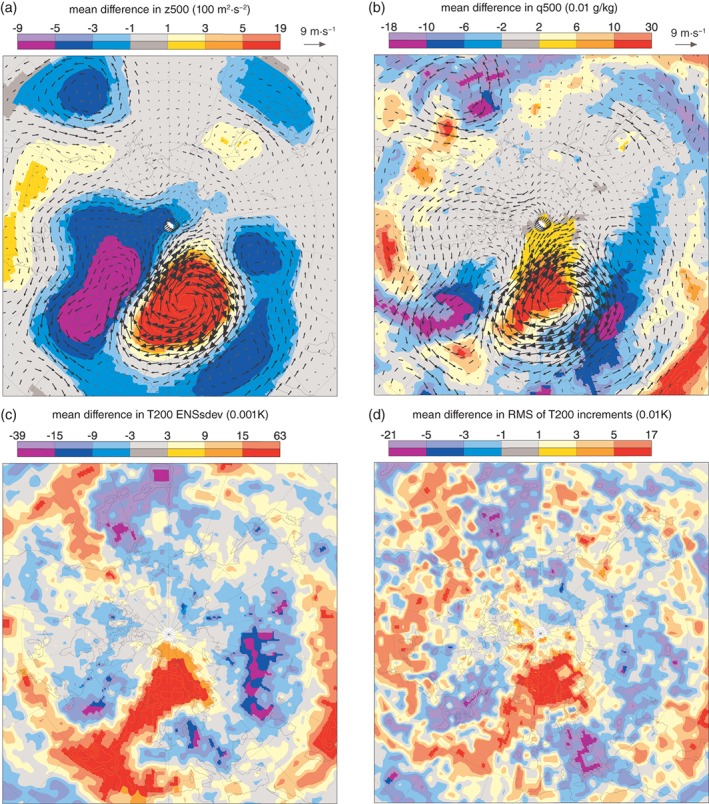
Average difference in (a) geopotential and (b) specific humidity, at 500 hPa for periods of Scandinavian blocking compared to “normal” conditions in ERA5 (DJFM 2013–2018). (c) As (a) but for temperature at 200 hPa (T200) EDA ensemble standard deviation. (d) As (a) but for the RMS of T200 analysis increments. Deep colours indicate that the differences are significant at the 95% level [Colour figure can be viewed at http://wileyonlinelibrary.com].

To begin, we separated the ERA5 forecast start times into two groups: a Scandinavian Blocking group and a “normal” group. These were selected based on whether the forecast start time was in the highest and middle tercile of the Scandinavian Blocking index introduced in section 3.2, calculated for all start times throughout the six seasons. The difference in the mean *z*500 of these two groups is clearly very similar (particularly over Europe) to the composites of cases where the Arctic has a strong influence on the midlatitudes shown in section 3.2 (Figures 3 and 8a). The corresponding humidity anomaly shows how intrusions of moisture into the Eurasian‐Arctic sector go hand‐in‐hand with this flow regime, consistent with the two case‐studies from the previous section.

The blocking pattern bears a strong resemblance to that shown by Sato *et al*. (2014), who found that this Scandinavian Blocking pattern, which is associated with anomalous warmth in the Eurasian‐Arctic was in part triggered by diabatic heating anomalies in the Gulf Stream region of the North Atlantic, which is consistent with the findings of Grams *et al*. (2018).

### 4.1 Initial errors

In the following we examine how the analysis increments and the variance in the ensemble of perturbed data assimilation (EDA: Isaksen *et al*., 2010; Bonavita *et al*., 2012) in ERA5 depends on this regime.

One way to diagnose error growth at short lead times (12 h) is to examine the “EDA ensemble variance” (EnsVar), and “analysis increments” from the data assimilation system that was used to create the initial conditions of the forecasts (Rodwell *et al*., 2016). The EnsVar is an estimate of the background error variance, while the “analysis increments” are what is added to the short‐range forecast (or the first guess) to obtain the analysis during the assimilation process. During episodes of high error growth, the departure of the first guess from the observations, and therefore the increments needed to “pull” the analysis toward the observations will be larger, as will the EnsVar.

We examine the differences in temperature increments and EnsVar (at 200 hPa) between the Scandinavian Blocking cases and the average conditions (Figure 8b,c). Note that the 200 hPa level was chosen because there are many more *in situ* observations at this level, as it is the approximate cruising altitude of commercial airliners. Both the analysis increments and the EDA‐Var are indeed larger in the European Arctic during periods of Scandinavian Blocking, compared to normal conditions (Figure 8b,c). Over Europe, the sign of these differences is negative, suggesting that errors are growing much less rapidly there due to stable conditions within the region of high pressure (Figure 8b). Further decomposition of the RMS of the analysis increments into mean and standard deviation components shows that the standard deviation, or random error component, is dominant here (not shown), rather than changes in the mean error, which suggest that these errors highlight differences in predictability between the two groups of start dates.

### 4.2 Influence on medium‐range skill over Asia

**Figure 9:**
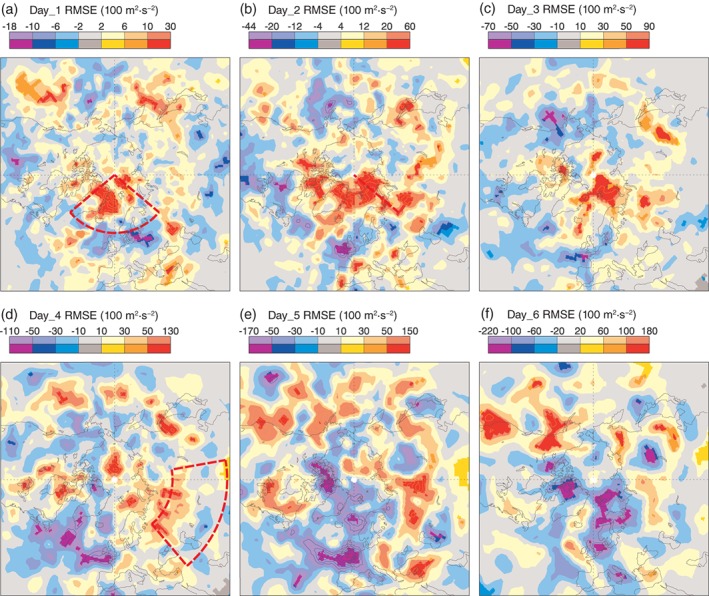
Difference in *z*500 RMS error in forecasts initialised during Scandinavian Blocking episodes, compared to average conditions (DJFM 2011–2018). Verification is performed against the ERA5 analysis fields. Deep colours indicate that the differences are significant at the 5% level. Red boxes show regions used in the correlation analysis [Colour figure can be viewed at http://wileyonlinelibrary.com].

Given the connection between errors in the European Arctic and north Asia shown in section 3, it seems likely that such increased errors in the Arctic at short range would also lead to downstream errors over north Asia at longer lead times. Indeed, analysis of the *z*500 RMSE in the 10‐day forecasts initialised from ERA5 show that at short ranges (days 1–2) the errors are larger in the Arctic itself during Scandinavian Blocking events, as one would expect based on the analysis of increments (Figure 9). At days 4 and 5, the area of increased errors has spread over northern Asia (see Figure 9), in an area which broadly corresponds to the area of high Arctic impact identified in section 3.1 (Figure 1). The relationship between the errors in the European‐Arctic and over north Asia (shown in Figure 9) can also be seen in the correlation of *z*500 RMSE for the two regions at different times of the forecast. The Pearson‐r correlation between the RMSE at day 4 in north Asia and day 1 in the Arctic, although small (*r* = 0.22, *p* = 3 × 10^−6^), is highly significant for forecasts initialised during Scandinavian Blocking periods. During non‐blocking periods this drops (*r* = 0.11, *p* = 0.02) and the difference between the two is significant at the 5% level using a Fisher r‐to‐z transformation.

These results corroborate the main findings from the OSE and relaxation experiments by demonstrating that the European‐Arctic has an increased sensitivity to the initial conditions during episodes of Scandinavian Blocking. It also shows that the magnitude of short‐range forecast error in the European‐Arctic and medium‐range error in northern Asia are causally linked during Scandinavian Blocking episodes. Further, it shows that these findings of section 3, which focused on a specific year and specific cases, are a general feature. Returning to the interpretation of the OSEs: if, for example, the IN‐SITU Arctic observations had not been used in ERA5 (i.e. if one ran an Arctic OSE with the ERA5 system for this period), the differences in the integrated error growth in this region, and the downstream errors in north Asia would be even larger during Scandinavian Blocking episodes.

## 5 CONCLUSIONS

In this study, we used three approaches to understand the influence of the Arctic on medium‐range weather forecasts in north Asia, and how this impact depends on flow type during the winter season. The central questions were: (a) does the Arctic observing system have an impact on the skill of medium‐range weather forecasts in north Asia in all cases, or does the strength of the influence vary as a result of changes in flow regime, (b) are insights about the regime dependence of Arctic‐to‐midlatitude linkages, gained from the Arctic OSEs, consistent with those derived from relaxation experiments? And (c) to what extent are they also reflected in the climatology of forecast errors?

In summary, we found that during boreal winter:

1. The degradation of the initial conditions in the Arctic, induced by removing Arctic *in situ*, microwave and infrared satellite observations when creating the initial conditions of the forecasts, leads to a decrease in forecast skill over northern Asia in the medium‐range. A broadly similar pattern, of albeit opposite sign, is found when the Arctic is relaxed toward our best estimate of the atmospheric state in relaxation experiments. However, in general the impact of the relaxation on midlatitudes is more widespread and typically larger than the impact found in the OSEs.
2. The medium‐range impact in north Asia, induced either through the Arctic observing system or relaxation experiments, is largest during episodes of Scandinavian Blocking. This is because:
  - Short‐range error growth in the European‐Arctic is larger than average during Scandinavian Blocking episodes, when warm‐moist intrusions lead to higher baroclinicity in the region. As a result, the sensitivity to removing observation when creating the initial conditions is larger then.
  - The high‐amplitude stationary waves that occur during Scandinavian Blocking episodes allow efficient propagation of forecast errors out of the European‐Arctic into north Asia.
3. Analysis of medium‐range forecasts initialized from ERA5 (for the period 2013–2018) show that points 2a and 2b are general features of the forecast error climatology over a longer period.

The results from this study provide some guidance on how, when and where improving the representation of the Arctic, for example through improvements to the observing system or the use of observations in NWP, would improve forecasts in the midlatitudes during winter. By comparing Arctic relaxation experiments and OSEs for the first time, the study also allows us to interpret the results from relaxation experiments, which have been used recently to understand the influence of the Arctic on midlatitudes from a forecast perspective. The relaxation experiments provide an upper bound of the forecast improvement that one would get in the midlatitudes by improving the representation of the Arctic because they limit growth of errors in the initial conditions as well as errors arising from forecast model error. As a result, they cannot be directly used to inform of the design or better use of the observing systems in the same way as the OSEs. However, our comparison shows that they do provide a consistent picture on how the Arctic‐to‐midlatitude linkages are modulated by the Scandinavian Blocking regime, suggesting that they could also be used to identify other situations in which improvements in the initial conditions, or other aspects of forecast, in the Arctic might improve forecasts in midlatitudes.

The idea that the influence of the Arctic on midlatitudes is flow‐dependent was recently proposed in the study of Semmler *et al*. (2018). This study goes further and shows that the periods where the Arctic has a strong influence on north Asia are also periods where the midlatitudes have a strong influence on the Arctic. In particular, during Scandinavian Blocking episodes the crests of planetary waves extend into the Arctic causing high baroclinicity and associated rapid error growth but also a mechanism for errors to be propagated out of the Arctic as well. Although this study has focused on such patterns over Eurasia, it is possible that similar high‐amplitude planetary waves in the Pacific–North‐American sector would lead to a similar influence over North America. Further, if planetary‐scale waves elongate meridionally and slow down in response to climate change, as has been suggested (Francis and Vavrus, 2012), then it is plausible the influence of the Arctic on midlatitudes on synoptic time‐scales may become more pronounced.

A corollary of our findings is that increasing the observational coverage in regions of high error growth in the European Arctic during Scandinavian Blocking events should improve forecast errors, not just in this part of the Arctic, but also downstream over north Asia. Indeed, such flow‐dependent error growth suggests that a more dynamic observing network, where more observations are taken in regions where error growth is fast might be advantageous.
